# Determination of sulfonamides in milk by capillary electrophoresis with PEG@MoS_2_ as a dispersive solid-phase extraction sorbent

**DOI:** 10.1098/rsos.172104

**Published:** 2018-05-23

**Authors:** Jianxin An, Xuan Wang, Meiting Ming, Jian Li, Nengsheng Ye

**Affiliations:** 1 Department of Chemistry, Capital Normal University, Beijing 100048, People's Republic of China; 2 Beijing Institute of Veterinary Drugs Control, Beijing 102206, People's Republic of China

**Keywords:** capillary zone electrophoresis, dispersive solid-phase extraction, milk sample, molybdenum disulfide, polyethylene glycol, sulfonamide

## Abstract

A synthetic polyethylene glycol-molybdenum disulfide (PEG@MoS_2_) composite was prepared using a simple method, and the application of this material in dispersive solid-phase extraction (DSPE) was investigated for the enrichment of eight sulfonamides (SAs) in milk samples. The composite was characterized by energy dispersive spectroscopy, scanning electron microscopy, transmission electron microscopy, Fourier transform infrared spectroscopy and Brunauer–Emmett–Teller measurements. The results showed that the MoS_2_ synthesized in the presence of PEG has the advantage of a larger surface area and that the adsorption effect of this MoS_2_ was enhanced. After extraction, the eight SAs were separated by capillary zone electrophoresis with a good linear relationship (*R*^2^ > 0.9902) in the range of 0.3–30 µg ml^−1^ and good precision (between 0.32% and 9.83%). Additionally, good recoveries (between 60.52% and 110.91%) were obtained for the SAs in the milk samples. The developed PEG@MoS_2_-based DSPE method could be applied for the enrichment of SAs in real milk samples.

## 1. Introduction

MoS_2_ is a typical transition metal disulfide with a layered structure, in which the molybdenum atoms are sandwiched between two layers of sulfide atoms. The molybdenum and sulfide atoms are covalently bonded together, and S-Mo-S interacts through van der Waals forces [1]. The applications of MoS_2_ have mainly focused on energy storage and transformation [2], phototransistors [3], catalysis [4], biosensors [5,6], cancer therapy [7,8] and analytical science [9–13]. Additionally, it also has good application prospects in lithium-ion batteries [14,15]. In the field of sample preparation, dahlia-like MoS_2_ nanostructures can pre-concentrate and extract heavy metals [9]. The MoS_2_ nanosheets have numerous sulfide atoms on their surfaces and edges, and these sulfide atoms can form complexes with metal ions, allowing them to concentrate and extract the target ion via electrostatic adsorption. MoS_2_ and functionalized multiwalled carbon nanotubes have been prepared as composite materials for the modification of electrodes, and the chloramphenicol concentrations in milk, honey and powdered milk were successfully determined with such an electrochemical sensor [11]. It had been suggested that polyvinyl alcohol can be used to prepare MoS_2_ nanocomposites to improve their heat and endurance performance [16], and now it was suggested that with the help of non-ionic surfactants, such as polyethylene glycol (PEG), MoS_2_ microspheres were synthesized via a hydrothermal method, and the spheres can adsorb methylene blue and organic pollutants in aqueous solutions [17]. To enhance the applicability of MoS_2_ in solid-phase extraction (SPE), MoS_2_ was combined with reduced graphene oxide to form hybrid nanosheets to improve the extraction of Pb (II) and Ni (II) [12]. Recently, magnetic MoS_2_-Fe_3_O_4_ nanocomposites were synthesized and used for the dispersive solid-phase extraction (DSPE) of Pb (II) and Cu (II) in water and plants [13].

Sulfonamides (SAs) are widely used in the treatment and prevention of human and animal infections caused by microbial agents. As SAs have a wide range of activities and are inexpensive, they are widely used in veterinarian clinical practices. The improper use of SAs can lead to residues in animal tissues or animal-derived foods. Therefore, the determination of SA residues is very important, and many methods have been developed for the analysis of SAs [18]. The commonly used analytical methods for SAs include high-performance liquid chromatography (HPLC) [19–25], ultra-performance liquid chromatography (UPLC) [26,27] and capillary electrophoresis (CE) [28–34]. Owing to the low concentration levels of SAs in real samples, some new sample preparation methods have been developed for the enrichment and extraction of SAs [35–39]. For example, a simple and economical liquid–liquid extraction method combined with high-performance liquid chromatography–diode array detection was developed for the determination of seven SAs commonly found in chickens and eggs [35]. By another method, a new type of automatic online SPE procedure coupled with CE, was developed to analyse SAs in sewage [36]. To improve the speed and reduce the cost, a QuEChERS (quick, easy, cheap, effective, rugged and safe) and liquid–liquid micro-extraction method was used for the extraction of 19 types of SAs in pork samples, and an ultra-high-performance liquid chromatography with tandem mass spectrometry (UHPLC-MS/MS) method was used to achieve good linearity and recovery [37]. Some novel materials, including metal-organic frameworks and graphene-based materials, were used for the enrichment of SAs in different samples [19,21,38,39].

**Figure 1.**
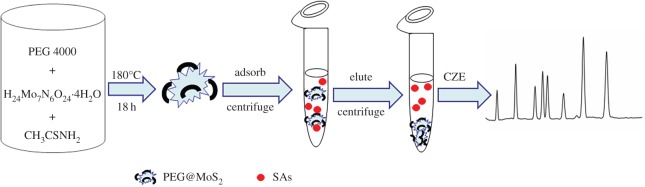
The flow chart of the synthesis of PEG@MoS_2_ and its application in the determination of SAs by DSPE-CZE.

In this report, PEG@MoS_2_ composites were synthesized and used as DSPE adsorbents for the pretreatment of SAs (sulfathiazole (STZ), sulfadimidine (SDD), sulfadiazine (SDZ), sulfamethoxazole (SMZ), sulfacetamide (ST), sulfachloropyridazine (SCD), N_4_-phthalylsulfathiazole (PST) and succinylsulfathiazole (SST)) based on the adsorption properties of MoS_2_ materials [40], and the SAs were then analysed by capillary zone electrophoresis (CZE). This developed method can be used to determine the SAs in milk samples, and the flow chart of this research is shown in figure 1.

## 2. Material and methods

### 2.1. Reagents

Ammonium molybdate tetrahydrate (H_24_Mo_7_N_6_O_24_·4H_2_O) (99%), thioacetamide (TAA) (98%), sulfathiazole (STZ), sulfadimidine (SDD), sulfadiazine (SDZ), sulfamethoxazole (SMZ), sulfacetamide (ST), sulfachloropyridazine (SCD), N_4_-phthalylsulfathiazole (PST), succinylsulfathiazole (SST), sodium hydrogen phosphate (Na_2_HPO_4_), sodium dihydrogen phosphate (NaH_2_PO_4_), methanol for HPLC (99.9%) and acetonitrile for HPLC (99.9%) were purchased from Sigma-Aldrich (St Louis, USA). Acetone, sodium hydroxide (NaOH) and hydrochloric acid (HCl) were purchased from Beijing Chemical Works (Beijing, China). Polyethylene glycol (PEG4000) was purchased from Merck (Germany). Ultrapure water (18.2 MΩ cm) was prepared using a Milli-Q Gradient ultrapure water system (Millipore, Milford, MA, USA).

The eight SAs were dissolved in a 0.1 mol l^−1^ NaOH solution to prepare standard stock solutions of 2.0 mg ml^−1^ for each SA, and these standard solutions were stored at 4°C and protected from light. The working solutions to be analysed were diluted with ultrapure water. The calibration standards of SAs were prepared with five levels of concentration in the range of 0.3 to 30 µg ml^−1^.

### 2.2. Apparatus

CZE was performed on a P/ACE MDQ high-performance CE system with a diode array detector (Beckman Coulter, USA). The 32 Karat software (Version 8.0) was used to control the CZE system and collect all of the experimental data. The capillary column was purchased from Yongnian Ruifeng Chromatographic Equipment (Hebei, China). Scanning electron microscopic (SEM) images were obtained using an S-4800 SEM system (Hitachi, Japan), and transmission electron microscopic (TEM) images were obtained using a JEM 1200EXA system (JEOL, Japan). The characterization of functional groups contained in PEG@MoS_2_ was performed using a TENSOR 27 FTIR system (Bruker, Germany), and its specific surface area and pore size are investigated by MicroActive for ASAP 2460 (Version 2.01, Shanghai, China).

### 2.3. Preparation of PEG@MoS_2_ materials

The PEG@MoS_2_ powder was synthesized using a previously reported method [41,42]. PEG4000 (0.5000 g) and 0.1766 g of H_24_Mo_7_N_6_O_24_·4H_2_O were dissolved in 20.00 ml of ultrapure water, and 0.1500 g of TAA was dissolved in 10.00 ml of ultrapure water. The two solutions were mixed together to form a uniform and stable system. The mixture was transferred to the reaction kettle, placed at 180°C for 18 h, removed and cooled to room temperature. After the water and supernatant were removed, a black solid material was obtained and washed with ultrapure ethanol several times. Finally, the material was freeze-dried in an oven for 24 h.

### 2.4. Pretreatment of the milk samples

Two millilitres of a milk sample purchased from a local market was mixed with 3.00 ml of acetonitrile in a centrifuge tube and then centrifuged at 10 000 r.p.m. for 5 min. The supernatant was washed in another centrifuge tube and dried to approximately 1.0 ml under nitrogen to remove the acetonitrile. Then, 5.00 ml of ultrapure water was added, and the solution was shaken and filtered through a 0.45 µm filter to obtain a clear solution. The solution was stored at 4°C in the dark.

### 2.5. Clean-up of the milk sample by the dispersive solid-phase extraction procedure

PEG@MoS_2_ was used as the adsorbent for DSPE, and the following procedure was used for the DSPE experimental operation. First, 0.0500 g of PEG@MoS_2_ was added into a centrifuge tube, and 1.00 ml of the pretreated solution of milk was added to form a uniform suspension at 2000 r.p.m. for 5 min. The supernatant was removed after centrifugation. Then, 1.00 ml of methanol was added, and the solution was vortexed at 2000 r.p.m. for 20 min. The eluate was then passed through a 0.22 µm nylon membrane. Finally, the eluate was dried with nitrogen at room temperature, and the residue was redissolved in 0.20 ml of methanol/water (1 : 1, v/v) for CZE.

### 2.6. Capillary zone electrophoresis separation

A fused silica capillary (75 µm i.d., 31.0 cm total length and 21.0 cm effective length) was used as the separation column. An 80 mM phosphate buffer solution (pH = 7.26) was used as the running buffer, and the separation temperature and applied voltage were 25°C and 18 kV, respectively. The sample was injected at a pressure of 0.5 psi for 5 s, and the detection wavelength was 254 nm.

Before the first run, the capillary column was rinsed with 0.10 mol l^−1^ NaOH for 30 min, 0.10 mol l^−1^ HCl for 20 min and ultrapure water for 20 min, respectively. Between each sample injection, the capillary column was washed for 2 min with ultrapure water, and the buffer solution was used for another 2 min.

## 3. Results and discussion

### 3.1. Characterization of the PEG@MoS_2_ materials

**Figure 2.**
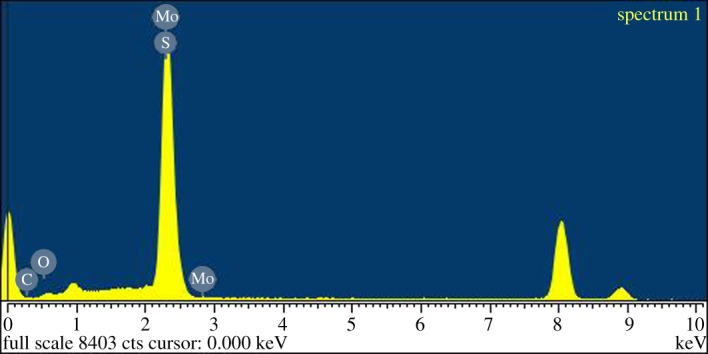
Energy dispersive spectroscopy of PEG@MoS_2_.

**Figure 3.**
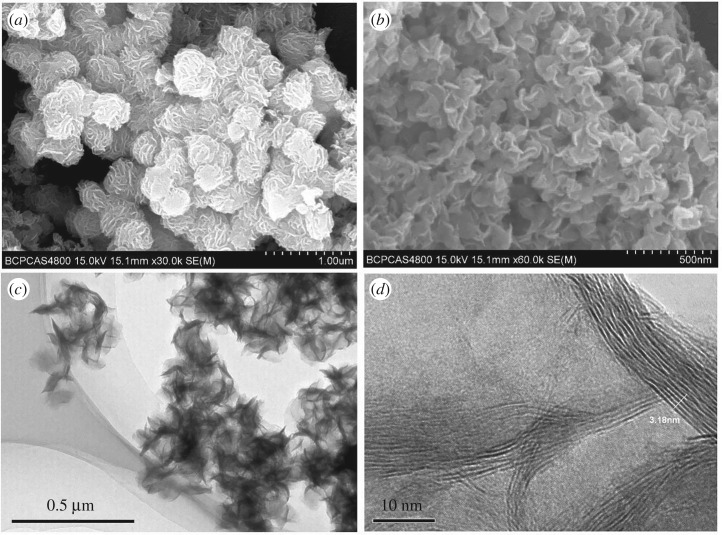
The SEM (*a*,*b*) and TEM (*c*,*d*) images of the PEG@MoS_2_ composites.

The PEG@MoS_2_ composites were characterized using energy dispersive spectroscopy (EDS), SEM, TEM, Fourier transform infrared spectroscopy (FTIR) and Brunauer–Emmett–Teller measurements (BET). According to the EDS (as shown in figure 2) analysis, the elements contained in the composites included C, O, Mo and S, proving that PEG and MoS_2_ were successfully combined. Figure 3 shows the SEM and TEM images of the PEG@MoS_2_ composites. The synthesized material has a cauliflower-like structure and microspheres with a diameter of approximately 400 nm. The surface is wrinkled and irregular with a multilayer structure and stacked layers, and the interlayer spacing is approximately 0.636 nm. This structure allows PEG@MoS_2_ to have a greater contact area with the target, which increases the contact sites and improves the adsorption capacity.

**Figure 4.**
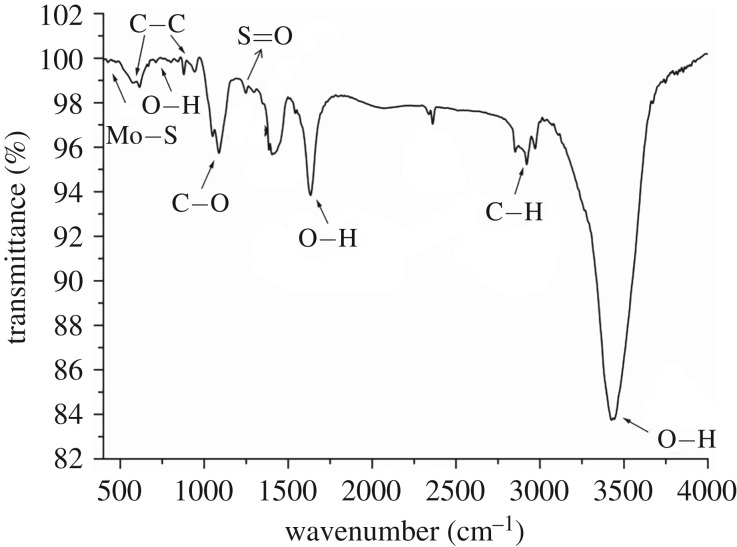
The FTIR spectrum of the PEG@MoS_2_ composites.

The chemical characteristics of the MoS_2_ composites were investigated using FTIR. Figure 4 shows the IR spectrum of the MoS_2_ layered structure [1,43]. The characteristic vibration peaks at 3427 and 1633 cm^−1^ may be the bending vibrations of the O-H in the water molecules adsorbed on the MoS_2_ composite. In addition, a weak extension in the region of 3000–2800 cm^−1^ was observed due to the stretching vibration of the methylene group, which indicated the adsorption of the PEG molecules on the MoS_2_ surface. The PEG molecules interact with water via hydrogen bonding to enhance the O-H vibrations. As shown in figure 4, the characteristic vibration peak appearing in the region of 500–400 cm^−1^ was attributed to the stretching vibration of the Mo-S bond in MoS_2_.

**Figure 5.**
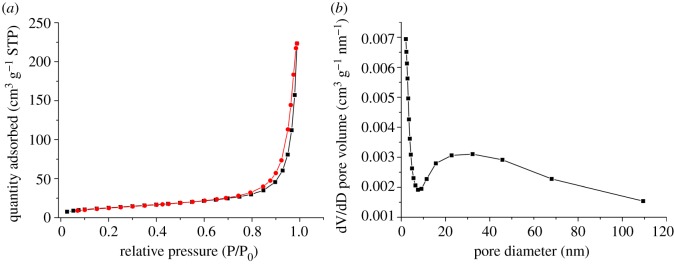
N_2_ adsorption–desorption isotherm (*a*) and the corresponding BJH pore size distribution (*b*) of the PEG@MoS_2_ composite.

The specific surface area and porosity of the PEG@MoS_2_ composites were investigated using a N_2_ adsorption analysis technique. As shown in figure 5, the specific surface area of the material was 46.54 m^2^ g^−1^, the average pore size was 29.69 nm and the total pore volume of the single point adsorption pore was less than 164.87 nm (P/P_o_ = 0.99 : 0.35 cm^3^ g^−1^). The MoS_2_ composites synthesized with PEG have a good specific surface area and pore size, which are beneficial for the adsorption of the target.

### 3.2. Optimization of the dispersive solid-phase extraction procedure

#### 3.2.1. Optimization of the eluent for the extraction of the sulfonamides

**Figure 6.**
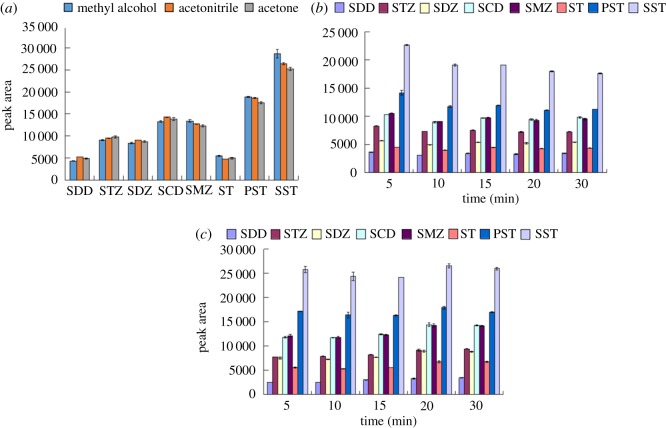
The effect of the elution solution (*a*), extraction time (*b*) and elution time (*c*) on the enrichment of SAs using the PEG@MoS_2_-based DSPE method.

A suitable solvent is necessary to elute the SAs from the adsorbent. In this study, three different organic solvents (methanol, acetonitrile and acetone) were used to elute the SAs from the PEG@MoS_2_ composites. Figure 6*a* shows the comparison of the elution of the eight SAs with different eluents, and methanol was selected as the eluent for subsequent experiments.

#### 3.2.2. Optimization of the extraction time and elution time for the dispersive solid-phase extraction procedure

The extraction time is one of the most important factors in the DSPE method. If the time is too short, the target cannot adsorb onto the material. If the time is too long, the material will strongly adsorb and take a long time to elute. Therefore, the choice of an appropriate extraction time is the key to the successful adsorption of matter. In this study, the adsorption after different extraction times (in the range of 5–30 min) was monitored. The results are shown in figure 6*b*. The adsorption capacity for the eight SAs gradually decreased as the time increased, and 5 min was chosen as the extraction time.

Based on the DSPE extraction time, the elution time was optimized over a range of 5–30 min. As shown in figure 6*c*, as the elution time increased, the SAs were reabsorbed by the sorbent. From the error bars, the 20 min elution time for more than half of the sulfonamides looks significantly better than the 15 min and 30 min elution times, and therefore, 20 min was chosen as the elution time.

### 3.3. Optimization of the capillary zone electrophoresis conditions

#### 3.3.1. Effect of the buffer concentration on the separation of sulfonamides

The buffer concentration has a significant effect on the separation results. In this study, the effect of the buffer concentration on the separation was investigated in the range of 20–100 mM. As shown in figure S1 in the electronic supplementary material, with increasing concentrations of the buffer, under the combined influence of the electro-osmotic flow and electrophoretic force, the separation effect gradually improved. As the buffer concentration increases, the friction between the buffer and the inner wall of the capillary column also increases, which will lead to increased temperature inside the column; therefore a buffer concentration of 80 mM was chosen as the optimum concentration for the subsequent experiments.

#### 3.3.2. Effect of the buffer pH on the separation of sulfonamides

The pH of the buffer solution also has a significant effect on the separation. As shown in figure S2 in the electronic supplementary material, as the pH value gradually increased, the separation between SDZ and SCD first increased and then decreased. The hydrogen on sulfonamido groups in sulfonamide molecules is easily dissociated by the sulfonyl electron-withdrawing action and appears weakly acidic. After dehydrogenation, the arylamino group is basic. At the same time, separation is influenced by the electro-osmotic flow. According to the calculated resolution, when the pH was 7.26, the separation between the two adjacent SA peaks was the best. Therefore, a pH of 7.26 was used for the follow-up experiments.

#### 3.3.3. Effect of the applied voltage on the separation of sulfonamides

In CZE, the voltage level has a significant impact on the ion mobility, which affects the peak time and separation efficiency. As shown in figure S3 in the electronic supplementary material, as the applied voltage increases, the speed of the electro-osmotic flow is affected, and as the migration time gradually decreases, ST and PST are separated. Considering the optimal use of the instrument and the separation effect optimization, 18 kV was chosen as the final separation voltage.

#### 3.3.4. Evaluation of the developed method

The performance results for the DSPE method with PEG@MoS_2_ are listed in tables 1 and 2. The eight SAs have good linear relationships (*R*^2^ = 0.9902–0.9969) in the range of 0.3–30 µg ml^−1^. The limits of detection (LODs) (S/N = 3) ranged from 0.03 to 0.20 µg ml^−1^. The intra-day precision was investigated for six parallel runs in the same day, and the relative standard deviations (RSDs) of the peak areas are in the range of 2.52–4.05% (*n* = 6). The inter-day precision was determined using a continuous three-day test, and the RSD values for the peak areas are in the range of 2.77% to 9.83% (*n* = 9).

**Table 1.** Analytical parameters of the PEG@MoS_2_-based DSPE method.

analyte	linear range (µg ml^−1^)	regression equation	*R*^2^	LODs (µg ml^−1^)
SDD	0.3–30	*y* = 1962.0*x* + 192.11	0.9949	0.16
STZ	0.3–30	*y* = 3661.24*x* + 1073.61	0.9958	0.04
SDZ	0.3–30	*y* = 3122.46*x* − 19.96	0.9939	0.03
SCD	0.3–30	*y* = 5276.34*x* − 7156.31	0.9928	0.10
SMZ	0.3–30	*y* = 4241.20*x* − 533.36	0.9969	0.07
ST	0.3–30	*y* = 1858.89*x* − 1152.68	0.9969	0.03
PST	0.3–30	*y* = 3324.62*x* + 6724.96	0.9902	0.20
SST	0.3–30	*y* = 6174.99*x* + 10177.74	0.9906	0.07

**Table 2.** RSD data of SAs by CZE with the MoS_2_-based DSPE method.

	intra-day precision (*n* = 6)		inter-day precision (*n* = 9)	
analyte	migration time	peak area	migration time	peak area
SDD	0.32%	3.80%	1.40%	7.70%
STZ	0.36%	3.31%	1.63%	9.83%
SDZ	0.46%	2.52%	1.90%	3.41%
SCD	0.56%	2.68%	1.98%	3.78%
SMZ	0.55%	2.54%	2.09%	2.77%
ST	0.55%	4.05%	2.31%	5.50%
PST	0.56%	3.51%	2.65%	9.76%
SST	0.67%	3.20%	3.00%	5.68%

### 3.4. Application of the PEG@MoS_2_-DSPE method to real samples

The developed method was used to determine the concentrations of eight SAs in a milk sample (as shown in figure S4 in the electronic supplementary material). The SAs were added to the prepared milk sample solution at concentrations of 0.3 µg ml^−1^ and 2.0 µg ml^−1^, and the solution was analysed using the previously proposed method (*n* = 3). A good recovery range of 60.52–110.91% was obtained, as listed in table 3. The recovery rates of the SAs showed that the milk sample composition had little effect on the enrichment of the SAs by the developed DSPE method.

**Table 3.** Recoveries from milk samples (*n* = 3).

analyte	real sample	concentration added (µg ml^−1^)	average recovery (%)	RSD (%)
SDD	ND	0.3	69.04 ± 0.16	0.28
		2	94.89 ± 2.40	3.09
STZ	ND	0.3	82.78 ± 0.79	1.18
		2	74.44 ± 1.43	2.71
SDZ	ND	0.3	66.96 ± 0.64	1.16
		2	75.09 ± 2.63	4.29
SCD	ND	0.3	91.75 ± 0.44	0.59
		2	60.52 ± 0.34	0.79
SMZ	ND	0.3	97.23 ± 0.28	0.35
		2	73.89 ± 2.51	4.16
ST	ND	0.3	110.91 ± 0.67	0.74
		2	82.32 ± 2.47	3.67
PST	ND	0.3	99.68 ± 2.00	2.46
		2	83.64 ± 5.16	8.72
SST	ND	0.3	61.80 ± 0.75	1.49
		2	105.06 ± 4.72	5.50

### 3.5. Comparison with previous methods

The proposed method is compared with the reported method for the detection of SAs. The results are presented in table 4. Compared with previous methods, PEG@MoS_2_ in the application of DSPE combined with CZE has good separation results. Compared with MSPE-HPLC and DLLME-HPLC, this method has lower LODs, and the MSPE-CE method has a wider linear range and better RSD. Thus, the proposed method can be used for the determination of SAs.

**Table 4.** An overview of existing methods for the determination of SAs. Note: sulfathiazole (STZ), sulfadimidine (SDD), sulfadiazine (SDZ), sulfamethoxazole (SMZ), sulfacetamide (ST), sulfachloropyridazine (SCD), N^4^-phthalylsulfathiazole (PST), succinylsulfathiazole (SST), sulfadimethoxine (SDM), sulfapyridine (SPD), sulfadoxine (SDX), sulfamethizole (SMI), sulfameter (SMT), sulfamethazine (SMN), sulfamerazine (SMR), sulfisoxazole (SIZ), sulfamethoxypyridazine (SMP).

analyte	sample preparation	method	matrix	linear range (µg ml^−1^)	LODs	recovery (%)	RSD (%)	reference
SDZ, SDD, STZ	MSPE	HPLC	environmental water	0.2–20	0.05–0.1 µg ml^−1^	67.4–119.9	0.04–9.0	[19]
SDZ, STZ, SMR, SMN, SMP	MSPE	HPLC-DAD	pork, chicken, shrimp	3.97–1000 ng g^−1^	1.73–5.23 ng g^−1^	76.1–102.6	<4.5	[23]
SPD, SDZ, SCD, SDX, SMX, SDM, SMI, SMT, SMN	DLLME	HPLC	milk	2.01 × 10^−3^–0.25	0.60–1.21 µg ml^−1^	90.8–104.7	2.9–9.7	[24]
SDZ, STZ, SMR, SMI, SCD, SMZ, SIZ, SDM	SPE	HPLC-UV	seawater samples	0.5–10	167 ng l^−1^	>75	<5	[25]
SDD, SDM, STZ, SDZ	SPE	CZE	meat	0.5–50	0.028–0.063 mg kg^−1^	60.9–111.4	2.5–3.4	[28]
SDX, SMR, SIZ, SMZ	MSPE	CE	milk	0.005–0.2	0.89–2.31 µg l^−1^	62.7–104.8	≤10.2	[29]
SDD, SDZ, STZ	Ag(III)-Lumol-SA	CE-CL	milk, pork, chicken	10–200, 2–50	0.65–3.14 µg ml^−1^	79.5–112.4	2.1–2.8	[32]
SMN, SMR, SDZ, SDM, SMZ, STZ	HF-LPME	CE-ED	real-world water	0.2–50, 0.2–100, 0.5–100	0.033–0.44 ng ml^−1^	75.1–109	0.2–4.9	[33]
SMN, SDZ, STZ	MWNT/C18SWNT/C18	CE	milk	—	0.03–0.069 mg l^−1^	98.8–103.2	5.4–8.2	[34]
SDD, STZ, SDZ, SMZ	DSPE/MoS_2_	CZE	environmental water	0.5–30, 0.5–50	0.05–0.12 µg ml^−1^	82.02–119.94	0.65–9.1	[40]
SDD, STZ, SDZ, SMZ, ST, SCD, PST, SST	DSPE/PEG@MoS_2_	CZE	milk	0.3–30	0.03–0.20 µg ml^−1^	61.80–110.91	0.32–9.83	this method

## 4. Conclusion

In this work, PEG was used in the synthesis of PEG@MoS_2_ to increase the adsorption area and adsorption performance of the MoS_2_ composite material, and the composite retained a layered stacked structure. The composite was used to separate SAs in milk samples, and its good adsorption capacity was highlighted. The results showed that PEG promoted the synthesis of microspheres of MoS_2_, thereby increasing its specific surface area and improving its adsorption effect. Most importantly, this work provides a wider range of options for sample pretreatment materials.

## Supplementary Material

Supplementary material

## Data accessibility

Electronic supplementary material is available online at https://dx.doi.org/10.6084/m9.figshare.5682865.
