# Supplementary material for: Determination of sulfonamides in milk by capillary electrophoresis with PEG@MoS2 as a dispersive solid-phase extraction sorbent
Source: R Soc Open Sci. 2018 May 23;5(5):172104. doi: 10.1098/rsos.172104 (PMC5990762; doi:10.1098/rsos.172104)
Supplement: Supplementary material [file rsos172104supp1.doc]

### Electronic Supplementary Material

### For

Determination of sulfonamides in milk by capillary electrophoresis with PEG@MoS2 as a dispersive solid-phase extraction sorbent

**Jianxin An^1^, Xuan Wang^1^, Meiting Ming^1^, Jian Li ^2^, Nengsheng Ye^1^***

1. Department of Chemistry, Capital Normal University, Beijing 100048, P. R. China.
2. Beijing Institute of Veterinary Drugs Control, Beijing 102206, People’s Republic of China

*CONTENTS*

***Figure S1***

*Effect of the buffer solution concentration on the separation of SAs.*

*Separation conditions: The separation voltage was 14 kV and the pH was 7.10.*

*Identification of peaks: 1-SDD; 2-STZ; 3-SDZ; 4-SCD; 5-SMZ; 6-ST; 7-PST and 8-SST.*

***Figure S2***

*Effect of the buffer solution pH on the separation of SAs.*

*Separation conditions: The buffer concentration was 80 mM and the separation voltage was 18 kV. Identification of peaks as Fig. S1.*

***Figure S3***

*Effect of the applied voltage on the separation of SAs.*

*Separation conditions: The buffer concentration was 60 mM and the pH was 7.10. Identification of peaks as Fig. S1.*

***Figure S4***

*A typical electropherogram of unspiked milk (a), milk spiked with 2.0 µg/mL of SAs (without DSPE) (b) and milk spiked with 2.0 µg/mL of SAs (with DSPE) (c). Identification of peaks as Fig. S1.*


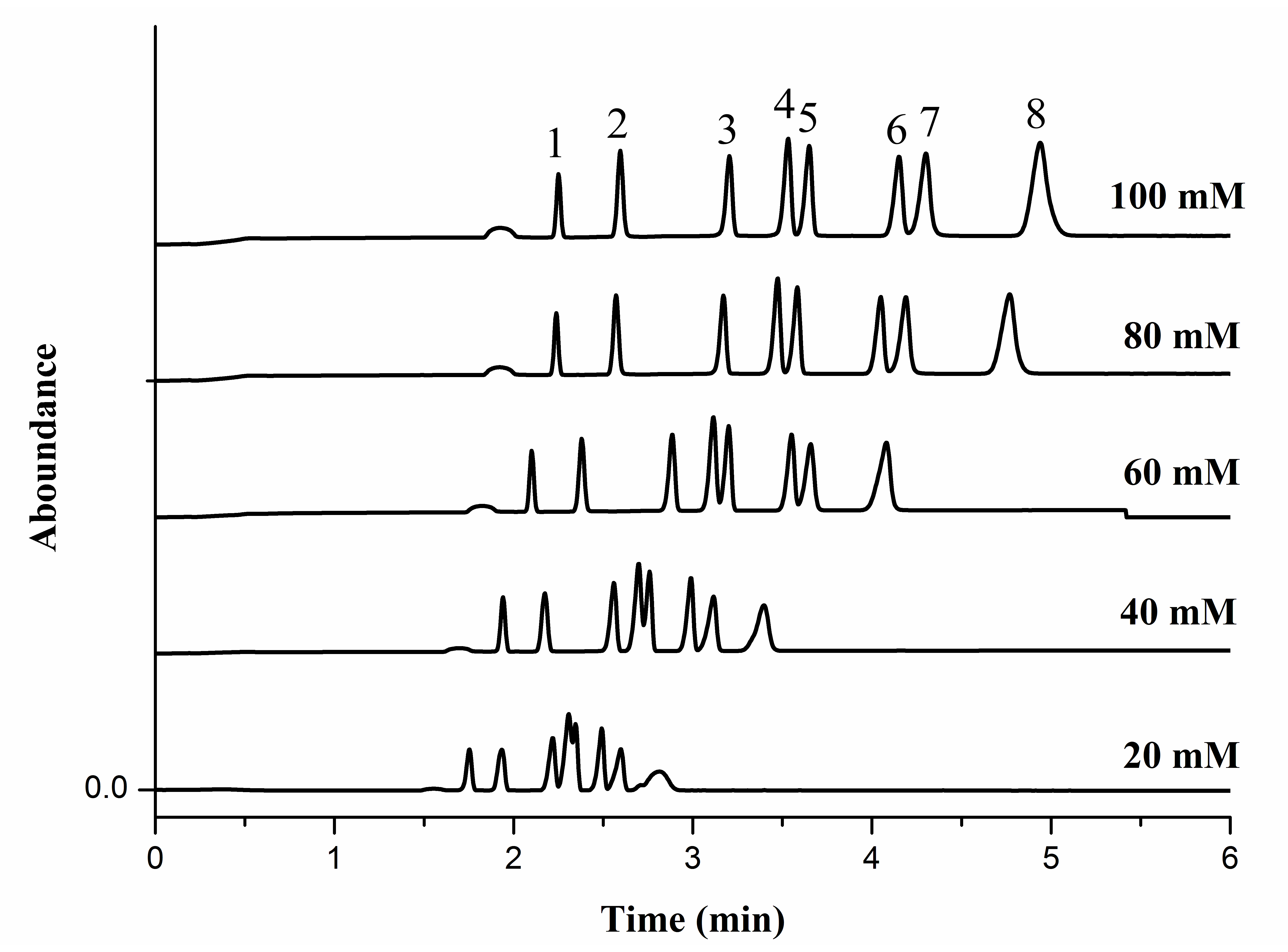


***Figure S1*** *Effect of the buffer solution concentration on the separation of SAs*

*Separation conditions: The separation voltage was 14 kV and the pH was 7.10.*

*Identification of peaks: 1-SDD; 2-STZ; 3-SDZ; 4-SCD; 5-SMZ; 6-ST; 7-PST and 8-SST.*


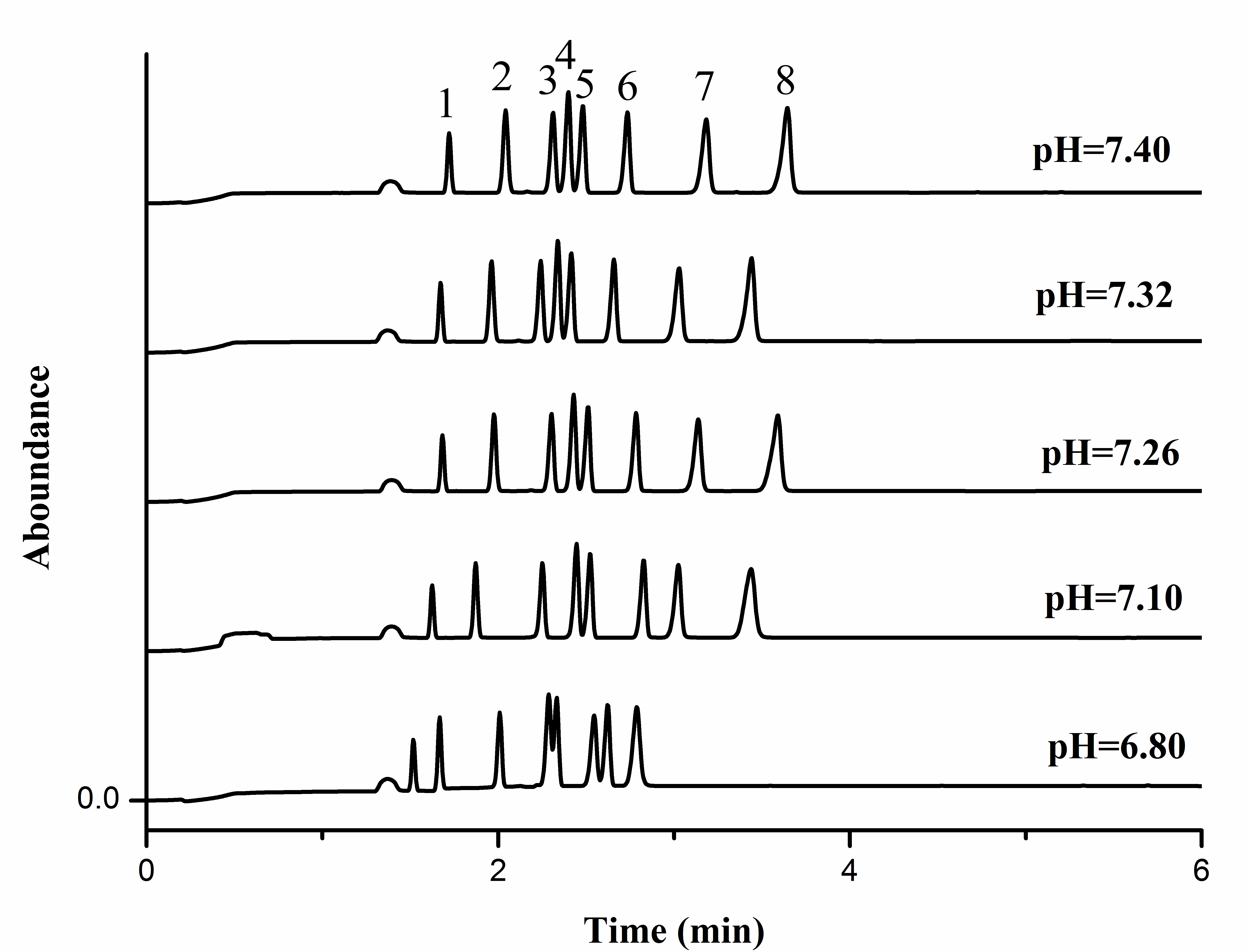


***Figure S2*** *Effect of the buffer solution pH on the separation of SAs*

*Separation conditions: The buffer concentration was 80 mM and the separation voltage was 18 kV. Identification of peaks as Fig. S1.*


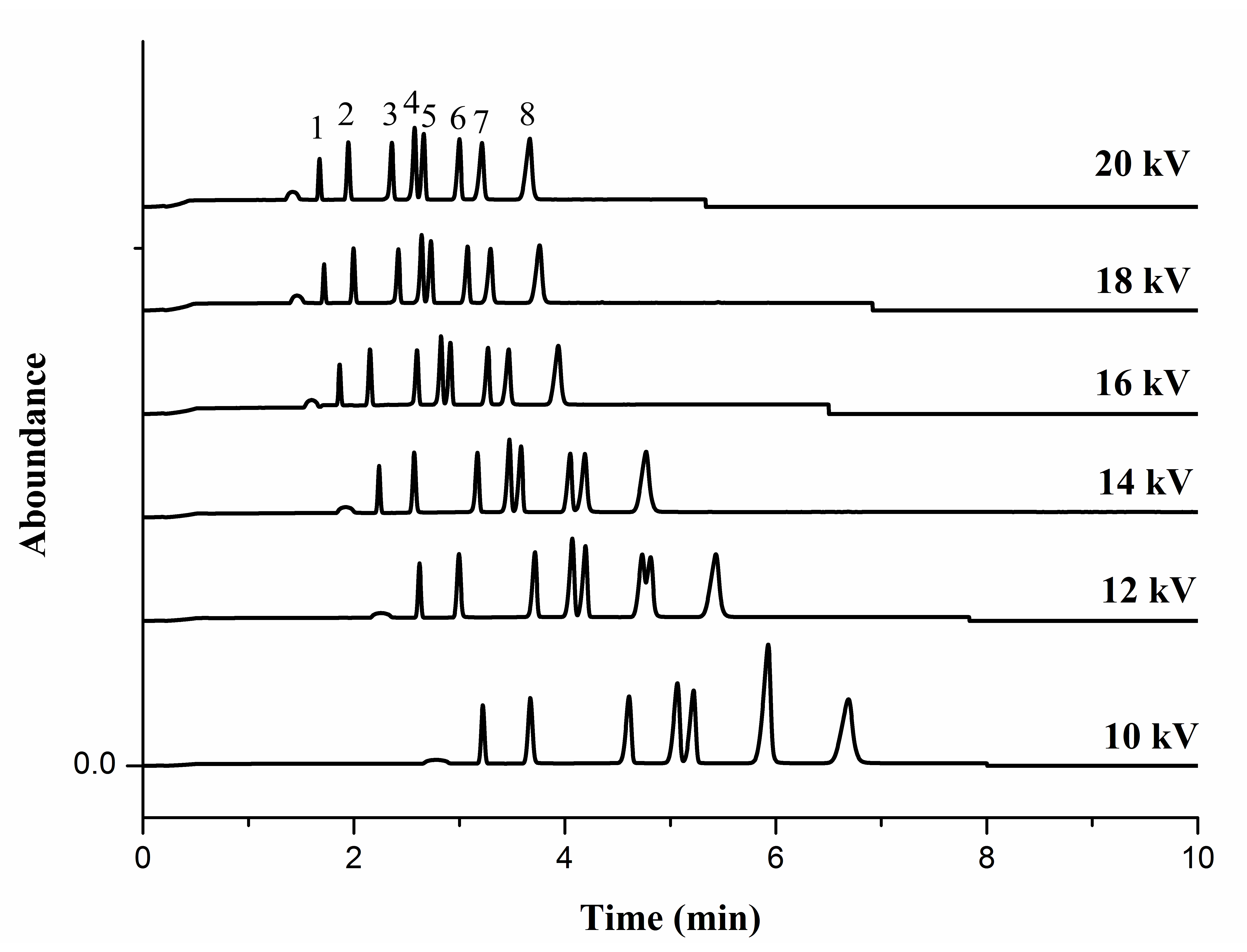


***Figure S3*** *Effect of the applied voltage on the separation of SAs*

*Separation conditions: The buffer concentration was 60 mM and the pH was 7.10.*

*Identification of peaks as Fig. S1.*

***Figure S4*** *A typical electropherogram of unspiked milk (a), milk spiked with 2.0 µg/mL of SAs (without DSPE) (b) and milk spiked with 2.0 µg/mL of SAs (with DSPE) (c). Identification of peaks as Fig. S1.*
